# *Yersinia pestis* in Small Rodents, Mongolia

**DOI:** 10.3201/eid1707.100740

**Published:** 2011-07

**Authors:** Julia M. Riehm, Damdindorj Tserennorov, Daniel Kiefer, Ingo W. Stuermer, Herbert Tomaso, Lothar Zöller, Dashdavaa Otgonbaatar, Holger C. Scholz

**Affiliations:** Author affiliations: Bundeswehr Institute of Microbiology, Munich, Germany (J.M. Riehm, D. Kiefer, L. Zöller, H.C. Scholz);; National Center for Infectious Diseases with Natural Foci, Ulanbaatar, Mongolia (D. Tserennorov, D. Otgonbaatar);; Institute for Zoology and Anthropology, Goettingen, Germany (I.W. Stuermer); Friedrich-Loeffler-Institut, Jena, Germany (H. Tomaso)

**Keywords:** Yersinia pestis, rodents, Mongolia, real-time PCR, CRISPR, Cardiocranius paradoxus, zoonoses, vector-borne infections, plague, letter

**To the Editor:** Plague is known to be endemic in several areas of Mongolia, but transmission to humans seems to play only a minor role because the number of recognized cases is relatively low (Figure) (*1*). The first human cases in Mongolia were reported to the World Health Organization in 1980, and <20 human cases have occurred each year since then (*2*). However, human plague was first reported in 1897 (*3*), such infections have been documented since the 1940s, and *Yersinia pestis* can be found in many provinces of Mongolia (Figure; T. Damindorj, pers. comm.) (*3**,**4*).

**Figure:**
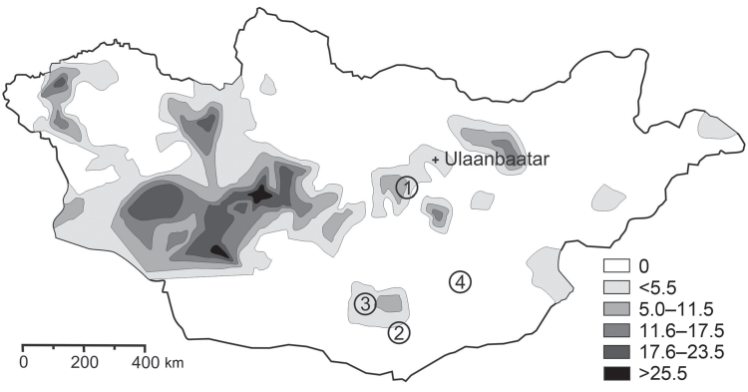
*Yersina pestis* in rodents in Mongolia. Shaded areas show the known distribution of enzootic plague in Mongolia during 1948–1999 (V. Batsaikhan, J. Myagmar, G. Bolormaa, National Center for Infectious Diseases with Natural Foci, Ulanbaatar, Mongolia; pers. comm.). The following 133 rodents were investigated: gerbils (*Meriones unguiculatus*, 61; *M. meridianus*, 25; *Rhombomys opimus*, 17); jerboas (*Allactaga sibirica,* 6; *Stylodipus telum*, 1; *Dipus sagitta*, 4; *Cardiocranius paradoxus*, 1), and squirrels (*Spermophilus alaschanicus*, 1; *Citellus dauricus*, 1). Plague-positive trapping loci were the following: 1, Tuv Aimag, Bayanunjuul Sum; 2–4, Umnugovi Aimag (2, Nomgon Sum; 3, Bayandalai Sum; 4, Manlai Sum). *Y. pestis* DNA was found in 7 rodents (gerbils and jerboas).

The most common source of human plague in Mongolia is contact with and consumption of the marmot (*Marmota sibirica*) (*1*). Moreover, the great gerbil (*Rhombomys opimus*) and the Mongolian gerbil (*Meriones unguiculatus*) are suspected of being enzootic reservoirs. Although small rodents are also assumed to be reservoirs of *Y. pestis*, the interaction of individual mammals or fleas of particular species in the infectious cycle and the dynamics of an epizootic are not yet clear (*5*). In a retrospective study, we screened tissue samples from small rodents for *Y. pestis* DNA to investigate the prevalence of *Y. pestis* in a potential enzootic reservoir.

During the course of zoologic investigations in Mongolia during 2002, 2005, and 2006, 133 rodents (gerbils, jerboas, and squirrels) were trapped by standard methods (*5*), dissected, and cataloged (Figure). Documentation included species, sex, date and location of trapping, animal size (weight, length) and organ dimensions, as well as all pathologic findings. Although the trapped animals showed a high degree of parasitic infestation, signs of a severe infectious disease were not observed. After the dissection of animals, samples were conserved in 70% ethanol.

Subsequently, total DNA was extracted from alcohol-conserved spleen and liver tissue of 133 animals by using QIAamp DNA Mini Kit (QIAGEN, Hamburg, Germany), according to the manufacturer’s instructions. Screening was performed by using a real-time PCR targeting the *pla* gene of *Y. pestis* pPCP1, including a PCR inhibition control, as described (*6*). As positive control, the *Y. pestis* vaccine strain EV76 was used. As negative controls, we included tissues of 53 laboratory rodents, which were processed analogs, beginning with DNA extraction.

In the real-time PCR targeting the *pla* gene, 7 (5.3%) of 133 spleen tissue samples were positive for *Y. pestis*. In contrast, all liver samples and samples of laboratory rodents tested negative. Identification of several host species was supported by partial sequencing of the cytochrome b gene (*7*). The animals tested positive for plague were gerbils (*Meriones* sp., 1; *M. unguiculatus*, 2; *Rhombomys opimus*, 2) and jerboas (*Allactaga sibirica*, 1; *Cardiocranius paradoxus*, 1).

The identity of the 230-bp *pla* PCR fragment was confirmed by DNA sequencing, showing 100% similarity to the *pla* gene sequences deposited in the European Molecular Biology Laboratory nucleotide database. Molecular subtyping of the 7 *pla*-positive DNA samples was attempted by clustered regularly interspaced short palindromic repeats analysis, targeting the 3 loci YPa, YPb, and YPc, respectively. Also included was DNA originating from the above-mentioned negative control tissues. However, only 1 sample from the spleen of a *M. unguiculatus* gerbil found the YPb locus, which then was sequenced, and resulted in the spacer signature *b1-b2-b3-b4-b5′*. This signature is known from a *Y. pestis* biovar, Orientalis, that has been isolated from *Rattus flavipectus* rats in the plague focus of the Yunnan–Guangdong–Fujian provinces in the People’s Republic of China (*8*).

Detection of *Y. pestis*–specific DNA in wild rodents has been described. For instance, a wild rodent community in the eastern Sierra Nevada mountains in the United States was screened for plague by *pla*-specific real-time PCR; of 89 rodents, 1 chipmunk (1.1%) had positive results (*9*).

The permanent presence of *Y. pestis* in rodent communities in North America has led to smaller and more distant-living colonies of prairie dogs (*10*). Strikingly, in the present study, >5% of the screened rodents were found to carry *Y. pestis* DNA. This high number was unexpected for the investigated areas, which have had a low level of plague activity (Figure). To our knowledge, *Y. pestis* has also not yet been reported in Manlai Sum (district) in the Umnugovi Aimag (subdivision) (Figure) (*2**–**4*) nor has the presence of *Y. pestis* DNA in a *Cardiocranius paradoxus* jerboa.

Our findings emphasize that rodents play a role as zoonotic reservoirs of *Y. pestis* in Mongolia and that the actual prevalence of plague seems to be underestimated. The low population density in Mongolia explains the low amount of illness in humans. Further investigations should include the screening of rodent populations near the plague-positive loci. In addition, fleas and other parasites (and also predators of small mammals) should be studied. Mongolia is a key area of plague genesis and therefore is an ideal location for more detailed study of the role of rodents as epizootic and enzootic reservoirs of *Y. pestis*.
